# β-TrCP Restricts Lipopolysaccharide (LPS)-Induced Activation of TRAF6-IKK Pathway Upstream of IκBα Signaling

**DOI:** 10.3389/fimmu.2018.02930

**Published:** 2018-12-13

**Authors:** Jin Liu, Yukang Yuan, Jing Xu, Kui Xiao, Ying Xu, Tingting Guo, Liting Zhang, Jun Wang, Hui Zheng

**Affiliations:** ^1^Institutes of Biology and Medical Sciences, Soochow University, Suzhou, China; ^2^Jiangsu Key Laboratory of Infection and Immunity, Soochow University, Suzhou, China; ^3^Department of Respiratory Medicine, The Second Xiangya Hospital, Institute of Respiratory Disease, Central South University, Changsha, China; ^4^Department of Intensive Care Medicine, The First Affiliated Hospital of Soochow University, Suzhou, China

**Keywords:** ubiquitination, E3 ligase, β-TrCP, protein kinase D1, LPS

## Abstract

β transducin repeat-containing protein (β-TrCP) is a Skp1-Cul1-F-box ubiquitin ligase, which plays important roles in controlling numerous signaling pathways. Notably, β-TrCP induces ubiquitination and degradation of inhibitor of NF-κB (IκBα), thus triggering activation of NF-κB signaling. Here, we unexpectedly find that β-TrCP restricts TRAF6-IKK signaling upstream of IκBα induced by lipopolysaccharide (LPS). In LPS-Toll-like receptor 4 (TLR4) pathway, protein kinase D1 (PKD1) is essential for activation of TRAF6-IKK-IκBα signaling including TRAF6 ubiquitination, IKK phosphorylation and subsequent IκBα degradation. We found that LPS promotes binding of β-TrCP to PKD1, and results in downregulation of PKD1 and recovery of IκBα protein level. Knockdown of β-TrCP blocks LPS-induced downregulation of PKD1. Supplement of enough PKD1 in cells inhibits recovery of IκBα protein levels during LPS stimulation. Furthermore, we demonstrate that β-TrCP inhibits LPS-induced TRAF6 ubiquitination and IKK phosphorylation. Taken together, our findings identify β-TrCP as an important negative regulator for upstream signaling of IκBα in LPS pathway, and therefore renew the understanding of the roles of β-TrCP in regulating TLRs inflammatory signaling.

## Introduction

The protein kinase D (PKD) family is a group of serine/threonine protein kinases that are comprised of three members (PKD1/PKCμ, PKD2, and PKD3/PKCν) (1). PKD family members have been shown to be essential for various cellular signaling pathways including activation of NF-κB and MAPKs, cell motility and adhesion, gene expression, and ROS (Reactive oxygen species) generation (2–5). PKD family is also involved in regulation of immune and inflammatory responses. It has been reported that PKD2 promotes phosphorylation and degradation of interferon alpha receptor 1 (IFNAR1), thus restricting interferon-mediated antiviral immunity (6). In addition, increasing evidence demonstrates that PKD family plays important roles in regulating Toll-like receptor (TLR)-mediated inflammatory signaling (7, 8). Recent studies found that a PKC/PKD inhibitor is able to inhibit lipopolysaccharide (LPS)-TLR4-mediated p38 activation and TNF-α secretion (9). PKD1, but not PKD2 and PKD3, is essential for MyD88-dependent ubiquitination of TNF-α receptor-associated factor 6 (TRAF6), phosphorylation of IKK, and subsequent activation of NF-κB and MAPKs in TLRs signaling (7). However, in spite of many important effects of PKD family on numerous signaling pathways, how cellular PKD protein levels are regulated remains largely unexplored.

Ubiquitin-mediated proteolysis is indispensable in regulating cellular levels and stabilities of diverse proteins, and therefore plays crucial roles in controlling cell signaling and human disease progression (10). There are three types of enzymes which catalyze the conjugation of ubiquitin to its substrates: ubiquitin-activating enzyme (E1), ubiquitin-conjugating enzyme (E2), and ubiquitin ligases (E3). To date, more than 600 human ubiquitin E3 ligases have been identified. The ubiquitin E3 ligases are the key determiners for the substrate specificity. Among these ubiquitin E3 ligases, β transducin repeat-containing protein (β-TrCP) has been clearly described, and regulates many important signaling proteins (11–16). Notably, β-TrCP induces ubiquitination and degradation of inhibitor of NF-κB (IκBα), which results in NF-κB release from the IκBα-NF-κB complex, and finally triggers activation of NF-κB signaling (11, 12). Thus, β-TrCP is recognized as a positive regulator for NF-κB-related inflammatory signaling pathway.

In the present study, we unexpectedly found that β-TrCP negatively regulates TRAF6-IKK signaling upstream of IκBα induced by LPS. LPS is a kind of endotoxin secreted by Gram-negative bacteria. As an activation model of inflammation, LPS is widely used for studying the regulation mechanisms of inflammatory signaling pathways. LPS activates TLR4 signaling by recruiting MyD88 to form the TLR4/MyD88/IRAK receptor complex, which promotes PKD1 activation and subsequent TRAF6 ubiquitination (8). Ubiquitinated TRAF6 then leads to phosphorylation and activation of TAK1 and IKK. Activated IKK induces IκB phosphorylation, which recruits β-TrCP to target IκB for ubiquitination and degradation. Finally, NF-κB is released from the IκB-NF-κB complex and translocates into the nucleus in an activated status.

Here, we revealed that β-TrCP can also target PKD1, a signaling molecule upstream of IκBα in LPS signaling, and promotes PKD1 ubiquitination and degradation. We found that LPS promoted interaction between β-TrCP and PKD1, and results in downregulation of PKD1 and recovery of IκBα protein level. Knockdown of β-TrCP blocks LPS-induced downregulation of PKD1. Consistently, β-TrCP attenuates LPS-induced TRAF6 ubiquitination and IKK phosphorylation. Collectively, our findings uncover that β-TrCP acts as an important negative regulator for upstream signaling of IκBα in LPS signaling.

## Materials and Methods

### Cell Culture

Human embryonic kidney cells (HEK293T) and RAW264.7 cell lines were obtained from ATCC. All cells were cultured at 37°C under 5% CO_2_ in Dulbecco's modified Eagle's medium (DMEM; Hyclone) supplemented with 10% FBS (GIBCO, Life Technologies), 100 units/mL penicillin, and 100 μg/mL streptomycin. According to the manufacturer's recommendation, plasmids were transfected into cells by using Longtrans (Ucallm).

### Expression Constructs and Reagents

Plasmids encoding β-TrCP and HA-β-TrCP were gifts from Dr. Serge Y. Fuchs (University of Pennsylvania). Expression plasmids HA- or GST-tagged PKD1, PKD2 and PKD3 were generated using PCR amplified from a HEK293T cDNA library. HA-Ub, HA-Ub-R48K, and HA-Ub-R63K were described previously (17). Myc-His-PKD1(1–444) and Myc-His-PKD1(445–912) were generated using PCR amplified from HA-PKD1. Myc-PKD1-Δ(172–312) and Myc-PKD1-Δ(311–444) were generated using PCR amplified from Myc-His-PKD1(1–444). Shβ-TrCP were purchased from GENECHEM (Shanghai, China). All mutations were generated by QuickChange site-Directed Mutagenesis Kit (Stratagene). All the plasmids were confirmed by DNA sequencing. Cycloheximide, MG132 and LPS were purchased from Sigma.

### RNA Isolation and Real-Time PCR

Total RNAs were extracted from HEK293T cells using TRIzol reagent (Invitrogen). The cDNA was produced by reverse transcription using oligo (dT) and analyzed by quantitative real-time PCR (qPCR) with PKD1, β-actin primers using SYBR Green Supermix (Bio-Rad Laboratories). The primer sequences were as follows: PKD1, 5′-GCCAACAGAACCATCAGTCC-3' and 5′-CTCCAATAGTGCCGTTTCCG-3′; β-actin, 5′-ACCAACTGGGACGACATGGAGAAA-3′ and 5′-ATAGCACAGCCTGGATAGCAACG-3′. The relative expression of the target genes mRNA was normalized to β-actin mRNA. The results were analyzed from three independent experiments and are shown as the average mean ± SD.

### Immunoblotting and Immunoprecipitation

Immunoblotting and immunoprecipitation were performed as described previously (17, 18). Briefly, all cells were harvested on ice using lysis buffer. N-ethylmaleimide (10 mM) was added to the lysis buffer when protein ubiquitination was detected. Immunoprecipitation was performed using specific antibodies overnight on a rotor at 4°C. Equivalent quantity of proteins were subjected to SDS-PAGE followed by transferring to PVDF membranes (Millipore). Then membranes were blocked with 5% non-fat milk or 5% BSA, and incubated with the corresponding primary antibodies, followed by the respective HRP-conjugated Goat anti-mouse or Goat anti-rabbit (Bioworld) secondary antibodies. Immunoreactive bands were depicted using SuperSignal West Dura Extended kits (Thermo Scientific). The following antibodies specific for GST (1:2000, HaiGene, M0301), PKD1 (1:500, Santa Cruz, sc-639), β-TrCP (1:1000, Cell signaling, #4394), HA (1:2000, abcam, ab9110), K63Ub (1:500, Cell signaling, D7A11), tubulin (1:5000, Proteintech, 66031-1-Ig), β-actin (1:5000, Proteintech, 66009-1-Ig), Myc (1:1000, abmart, #284566), IκBα (1:2000, Cell signaling, #4814), TRAF6 (1:500, Santa Cruz, sc-8409), Ubiquitin (1:1000, Santa Cruz, sc-8017), IKK (1:1000, Cell signaling, #2682), and pS176/18-IKK (1:1000, Cell signaling, #2697) have been used.

### Cycloheximide Chase Assay

The half-life of proteins was determined by cycloheximide (CHX) chase assay. HEK293T cells were transfected with GST-PKD1, with or without shβ-TrCP in 6-well culture plates. Seventy two hours after transfection, cells were treated with DMSO or CHX (50 mg/mL) for 0, 6, 12 h. Furthermore, cells were harvested on ice and subjected to analysis by western blotting.

### LPS Stimulation

RAW264.7 cells were treated with LPS (2.5 μg/mL) for different times. Cells were then harvested on ice using lysis buffer containing 150 mM NaCl, 20 mM Tris-HCl (pH 7.4), 1% Nonidet P-40, 0.5 mM EDTA, PMSF (50 μg/ml), and protease inhibitor mixtures (Sigma) and subjected to analysis by immunoblotting or immunoprecipitation.

### CRISPR-Cas9-Mediated Genome Editing

Small guide RNAs targeting mus PKD1: 1^#^ (5′-GCCCAGTCCGCTGCTGCCCG-3') and 2^#^ (5′-GCCGCACTGGTCCCAGGGTC-3′) were cloned into the lentiCRISPRv2 vector and were transfected into HEK293T cells. Forty eight hours after transfection, the supernatant was used to infect RAW264.7 cells. Then the RAW264.7 cells were cultured under puromycin selection until further experiments.

### *In vivo* Ubiquitination Assay

To analyze ubiquitination of TRAF6 in LPS signaling, RAW264.7 cells were treated with LPS (2.5 μg/mL) for 0, 15, 30 min. Cells were harvested in lysis buffer containing 150 mM NaCl, 20 mM Tris-HCl (PH 7.4), 1% NonidetP-40, 0.5 mM EDTA, PMSF (50 μg/ml), N-ethylmaleimide (10 mM) and protease inhibitors mixtures (Sigma). Endogenous TRAF6 proteins were immunoprecipitated using a specific TRAF6 antibody and then subjected to ubiquitination analysis using a specific ubiquitin (Ub) antibody by western blotting.

### Statistical Analysis

Comparison between different groups was analyzed by using a two-tailed Student's *T*-test. All differences were considered that *p* < 0.05 represent statistically significant.

## Results

### β-TrCP Negatively Regulates Protein Levels of PKD1, but Not PKD2 and PKD3

**Figure 1:**
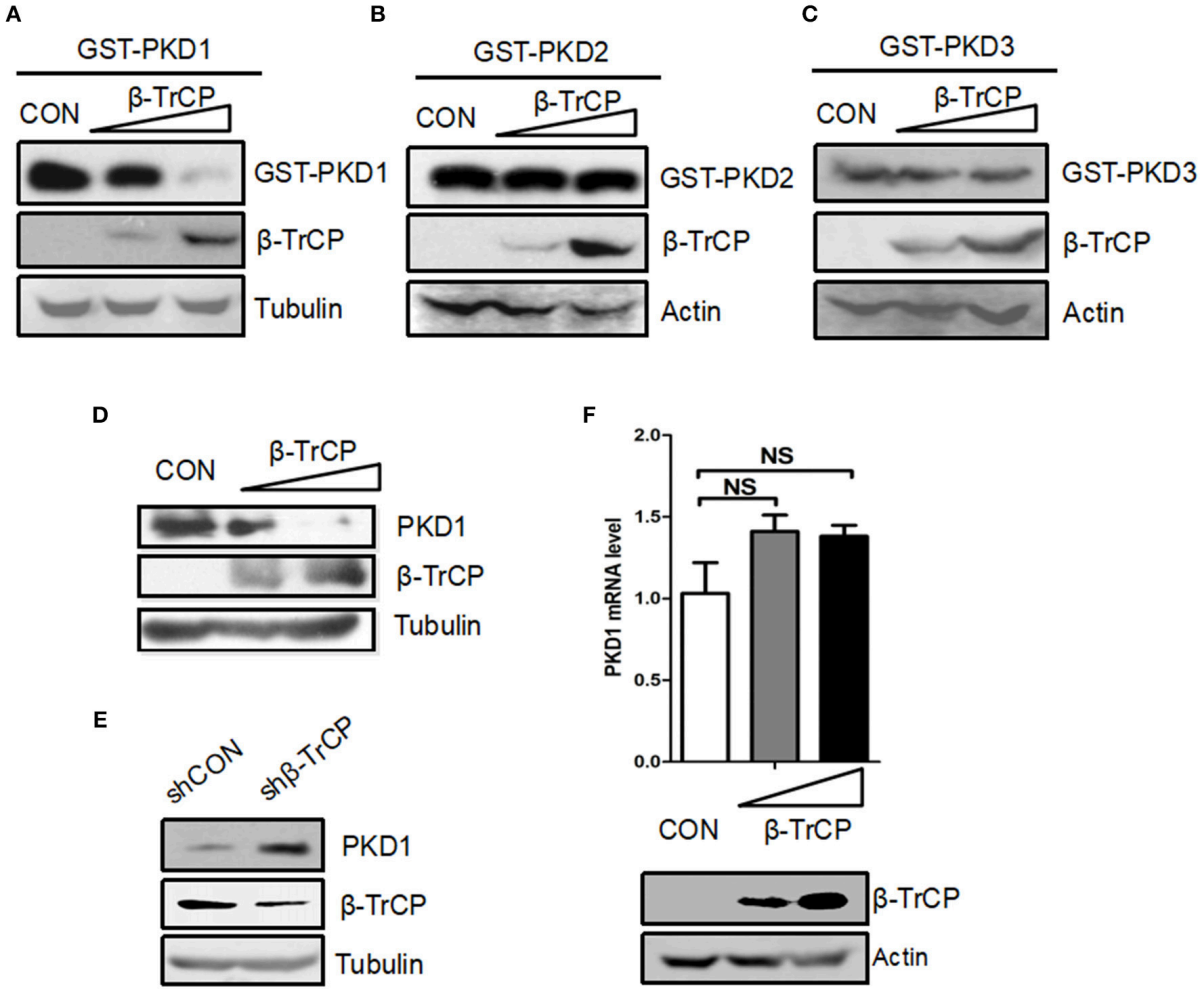
β-TrCP specifically promotes downregulation of PKD1. **(A–C)** HEK293T cells were transfected with either an empty vector or increasing doses of β-TrCP, together with GST-PKD1 **(A)** or GST-PKD2 **(B)** or GST-PKD3 **(C)**. Protein levels of GST-PKD1/2/3, β-TrCP and Tubulin were analyzed by immunoblotting as indicated. **(D)** HEK293T cells were transfected with increasing doses of β-TrCP. Protein levels of PKD1, β-TrCP, and Tubulin were analyzed by immunoblotting as indicated. **(E)** HEK293T cells were transfected with control shRNAs (shCON) or shRNAs against β-TrCP (shβ-TrCP). Protein levels of PKD1, β-TrCP and Tubulin were analyzed by immunoblotting as indicated. **(F)** HEK293T cells were transfected with empty vector or increasing doses of β-TrCP. The mRNA levels of endogenous PKD1 were measured by qRT-PCR. β-TrCP was analyzed by immunoblotting.

To explore the ubiquitin-mediated regulation of PKD family, we firstly analyzed the amino acid sequences of PKD family. We noted that PKD family members possess a putative DSG(X)_2+*n*_S motif, which has been demonstrated to be a characteristic of substrates for ubiquitin E3 ligase β-TrCP. Thus, we hypothesized that PKD family members could be potential substrates of β-TrCP. To determine the possible effect of β-TrCP on PKD family, we firstly analyzed the effects of β-TrCP overexpression on exogenous PKD family members. The results showed that overexpression of β-TrCP significantly downregulated protein levels of exogenous GST-PKD1 (Figure 1A), but not GST-PKD2 (Figure 1B) and GST-PKD3 (Figure 1C). To confirm the effect of β-TrCP on endogenous PKD1, we transfected cells with increasing amount of β-TrCP. We found that overexpression of β-TrCP gradually lowered protein levels of endogenous PKD1 (Figure 1D). Furthermore, endogenous β-TrCP was knocked down by shRNAs against β-TrCP (shβ-TrCP). We found that knockdown of β-TrCP upregulated protein levels of endogenous PKD1 in cells (Figure 1E). In addition, we found that overexpression of β-TrCP did not decrease PKD1 mRNA levels (Figure 1F), suggesting that β-TrCP regulates PKD1 at protein level. Taken together, our results suggest that β-TrCP is a negative regulator of cellular PKD1 protein.

### β-TrCP Promotes PKD1 Ubiquitination and Accelerates PKD1 Degradation

**Figure 2:**
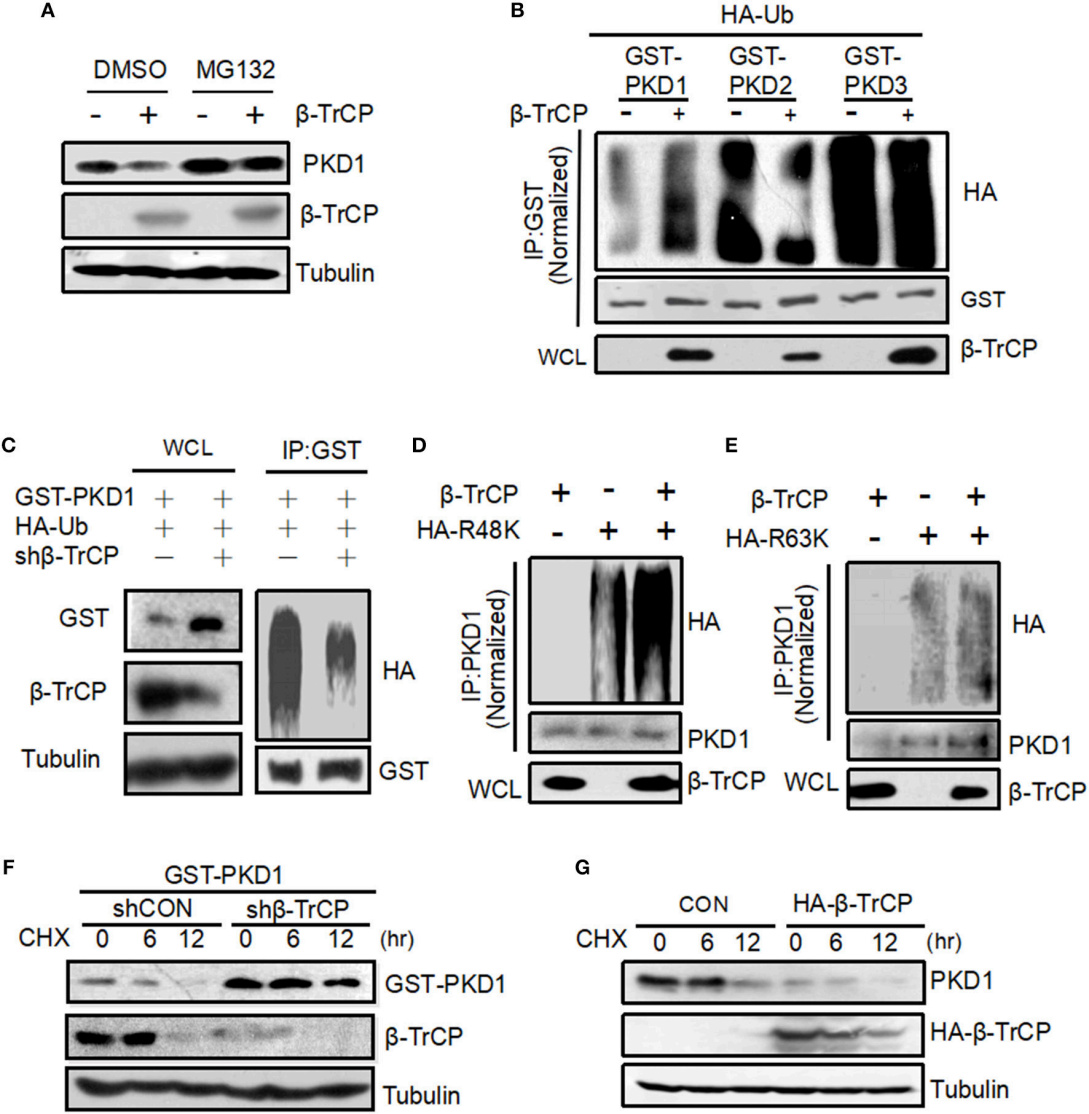
β-TrCP promotes PKD1 ubiquitination and accelerates PKD1 degradation. **(A)** HEK293T cells transfected with empty vector or β-TrCP were treated with or without MG132 (10 μM) for 12 h. Endogenous PKD1 levels were analyzed by immunoblotting as indicated. **(B)** HEK293T cells were transfected as indicated. GST-PKD1, GST-PKD2, and GST-PKD3 proteins were immunoprecipitated by a GST antibody. Ubiquitination and protein levels were analyzed using indicated antibodies. **(C)** HEK293T cells were transfected with shCON or shβ-TrCP, together with HA-Ub and GST-PKD1. Cells then were treated with MG132 (10 μM) for 4 h. Immunoprecipitation (IP) and immunoblotting (IB) were performed as indicated. **(D,E)** 293T cells were transfected with β-TrCP, together with HA-ub-K48 (R48K) **(D)** or HA-ub-K63 (R63K) **(E)** as indicated. PKD1 proteins were immunoprecipitated and then PKD1 ubiquitination was analyzed by indicated antibodies. **(F)** HEK293T cells were transfected with either shCON or shβ-TrCP, together with GST-PKD1. Seventy two hours after transfection, cells were treated with CHX (50 μg/mL) for 0, 6, and 12 h. Whole cell extracts were analyzed by immunoblotting using indicated antibodies. **(G)** HEK293T cells transfected with HA-β-TrCP. Forty eight hours after transfection, cells were treated with CHX (50 μg/mL) for 0, 6, and 12 h. Whole cell extracts were analyzed by immunoblotting using indicated antibodies.

To explore the mechanisms of PKD1 downregulation mediated by β-TrCP, we firstly took advantage of a proteasomal inhibitor MG132. Our results showed that MG132 inhibited β-TrCP-mediated PKD1 downregulation (Figure 2A), suggesting that β-TrCP promotes PKD1 downregulation via the proteasome pathway. Thus, we next analyzed whether β-TrCP is capable of regulating ubiquitination levels of PKD1. We found that overexpression of β-TrCP obviously promoted ubiquitination of GST-PKD1, but not GST-PKD2 and GST-PKD3 (Figure 2B), suggesting that β-TrCP specifically targets PKD1 member of PKD family. Furthermore, knockdown of β-TrCP upregulated PKD1 protein levels (Figure 2C, left panel) and significantly downregulated ubiquitination levels of PKD1 (Figure 2C, right panel). Next, we tried to determine whether β-TrCP induces K48-linked or K63-linked ubiquitination of PKD1. To this end, Ub-R48K (all lysines in Ub are mutated to arginines except lysine 48 residue) and Ub-R63K (all lysines in Ub are mutated to arginines except lysine 63 residue) were used to analyze the types of PKD1 ubiquitination. Our data showed that overexpression of β-TrCP significantly increased K48-linked polyubiquitination of PKD1 (Figure 2D). However, β-TrCP did not obviously affect K63-linked ubiquitination of PKD1 (Figure 2E).

Given that β-TrCP induces K48-linked ubiquitination of PKD1, we speculated that the protein stability of PKD1 could be regulated by β-TrCP. To address this hypothesis, we carried out cycloheximide (CHX) pulse chase analysis of PKD1 protein. Cells transfected with control vector (shCON) or shβ-TrCP were treated with protein synthesis inhibitor CHX. We found that knockdown of β-TrCP in cells substantially inhibited PKD1 degradation (Figure 2F), suggesting that β-TrCP knockdown enhances PKD1 protein stability. Furthermore, overexpression of β-TrCP accelerated the degradation of endogenous PKD1 protein (Figure 2G). In conjunction with the above observation showing that β-TrCP induces PKD1 K48-linked polyubiquitination, we think that β-TrCP can specifically promote PKD1 ubiquitination and degradation.

### NS_354_G Motif of PKD1 Is Required for β-TrCP Binding and Degradation of PKD1

**Figure 3:**
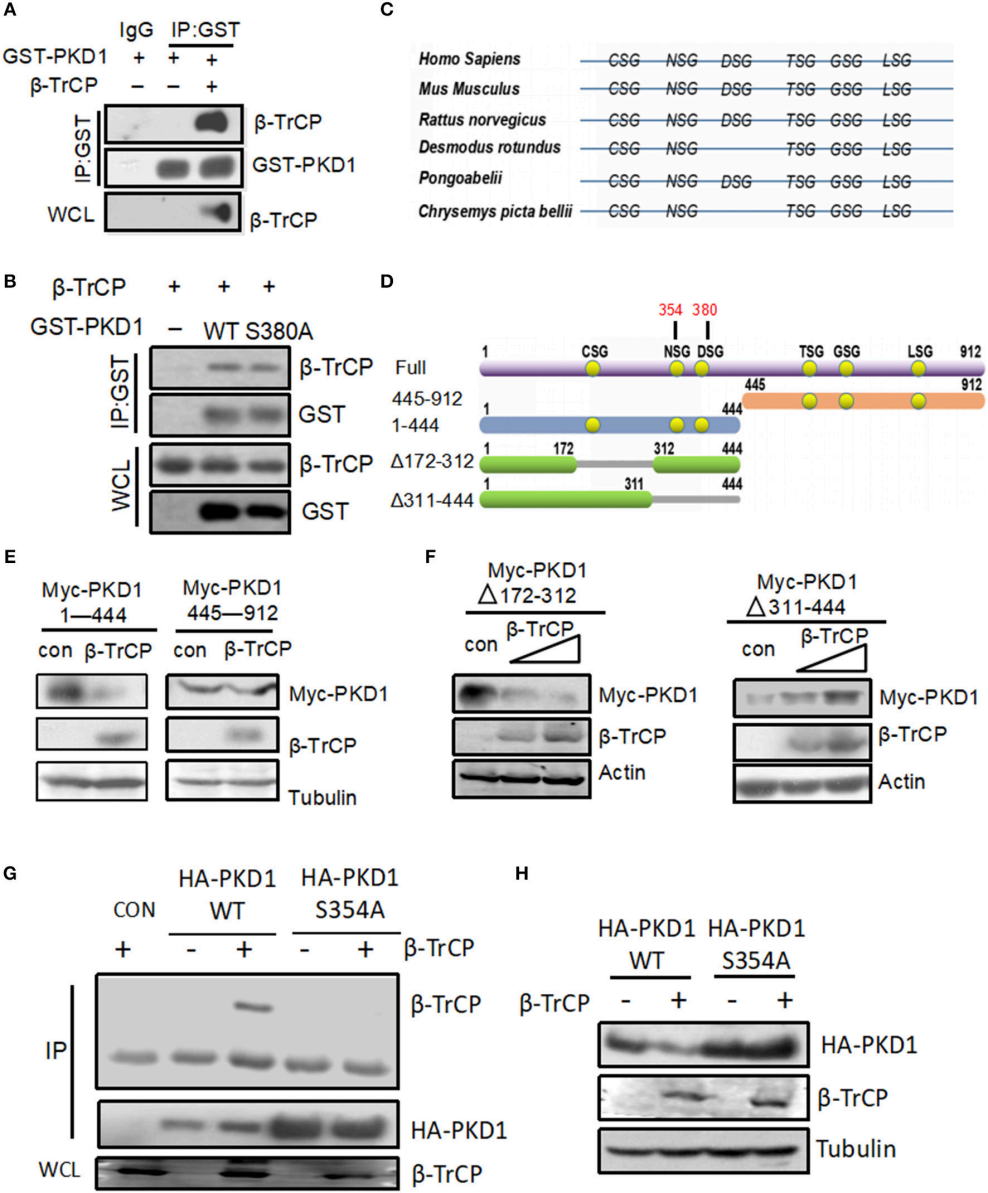
NSG motif of PKD1 is required for β-TrCP binding and degradation of PKD1. **(A)** HEK293T cells were transfected with GST-PKD1, together with or without β-TrCP. GST-PKD1 proteins were immunoprecipitated by a GST antibody, and protein levels of GST-PKD1, β-TrCP, and Tubulin were analyzed using indicated antibodies. **(B)** HEK293T cells were transfected with β-TrCP, together with GST-PKD1-WT or GST-PKD1-S380A. Whole cell lysates were subjected to IP-IB analysis as indicated. **(C)** XSG motifs in various species of PKD1. **(D)** Mapping of PKD1 full length and different mutants. **(E)** HEK293T cells were transfected with β-TrCP, together with either Myc-PKD1(1–444) or Myc-PKD1(445–912). Protein levels of Myc-PKDs mutants, β-TrCP and Tubulin were measured by immunoblotting with indicated antibodies. **(F)** HEK293T cells were transfected with either an empty vector or increasing doses of β-TrCP, together with Myc-PKD1-Δ(172–312) or Myc-PKD1-Δ(311–444). Immunoblotting was performed using indicated antibodies. **(G)** HEK293T cells were transfected with β-TrCP, together with HA-PKD1 (WT or S354A). Immunoprecipitation (IP) and immunoblotting were performed using indicated antibodies. **(H)** HEK293T cells were transfected with or without β-TrCP, together with HA-PKD1 (WT or S354A). Protein levels of HA-PKD1, β-TrCP, and Tubulin were analyzed by immunoblotting as indicated.

Previous studies have demonstrated that the conserved DSG(X)_2+n_S motif is an important characteristic of protein substrates for β-TrCP binding and subsequent protein substrates degradation. And mutation of the first serine (S) in this DSG(X)_2+n_S motif of a protein substrate will result in its inability to bind with β-TrCP. By analyzing the amino acid sequences of PKD1, we found that PKD1 does possess this DSG(X)_2+n_S motif. Therefore, we firstly determined the interaction between β-TrCP and PKD1. By co-immunoprecipitation assays, we found that β-TrCP is able to interact with PKD1 (Figure 3A). To our surprise, mutation of this serine 380 in DSG(X)_2+n_S motif of PKD1 did not obviously affect the binding of β-TrCP to PKD1-S_380_A (Figure 3B), indicating that β-TrCP interacts with PKD1 in an non-canonical manner.

We noticed that recent studies have reported several “atypical” substrates of β-TrCP, including STAT1 (19), Weel (20), GHR (21), ELAVL1/huR, CDC25B, and TP53 (22), which do not contain the classic DSG(X)_2+n_S motif. Instead, most of these atypical substrates have the XSG(X)_2+n_S motif. Therefore, we analyzed the potential XSG(X)_2+n_S motif in various species of PKD1 including Homo Sapiens, Mus Musculus, Rattus norvegicus, Desmodus rotundus, Pongoabelii, Chrysemys picta bellii (Figure 3C). To determine which XSG(X)_2+n_S motif of PKD1 is pivotal for binding with β-TrCP, we firstly constructed two PKD1 deletion mutants, PKD1 (1–444) and PKD1 (445–912) (Figure 3D). The ability of β-TrCP to downregulate two PKD1 mutants was analyzed. Our results showed that PKD1 (1–444), but not PKD1 (445–912), can be downregulated by β-TrCP overexpression (Figure 3E), indicating that the potential XSG(X)_2+n_S motif locates in PKD1 (1–444). PKD1 (1–444) possesses three conserved XSG(X)_2+n_S motifs (Figure 3D). Thus, we constructed another two PKD1 mutants, PKD1-Δ(172–312) and PKD1-Δ(311–444) (Figure 3D). We found that deletion of the 311–444 motif, but not the 172–312 motif, abolished β-TrCP-mediated downregulation of PKD1 (Figure 3F). These results suggested that the key XSG(X)_2+*n*_S motif locates in PKD1-Δ(311–444), which has two XSG(X)_2+n_S motifs, NS_354_G and DS_380_G. Our previous data have demonstrated that the DS_380_G motif of PKD1 is not essential for interaction between β-TrCP and PKD1 (Figure 3B). Therefore, we made a new PKD mutant, PKD1-S_354_A. Our data showed that mutation of serine 354 of PKD1 abolished the binding of β-TrCP to PKD1 (Figure 3G). Consistently, β-TrCP cannot downregulate PKD1-S_354_A protein, as compared with PKD1-wild type (WT) (Figure 3H). Taken together, we demonstrate that the NS_354_G motif of PKD1 is required for β-TrCP binding and PKD1degradation.

### LPS Promotes Binding of β-TrCP to PKD1 and Downregulates PKD1 Protein Levels

**Figure 4:**
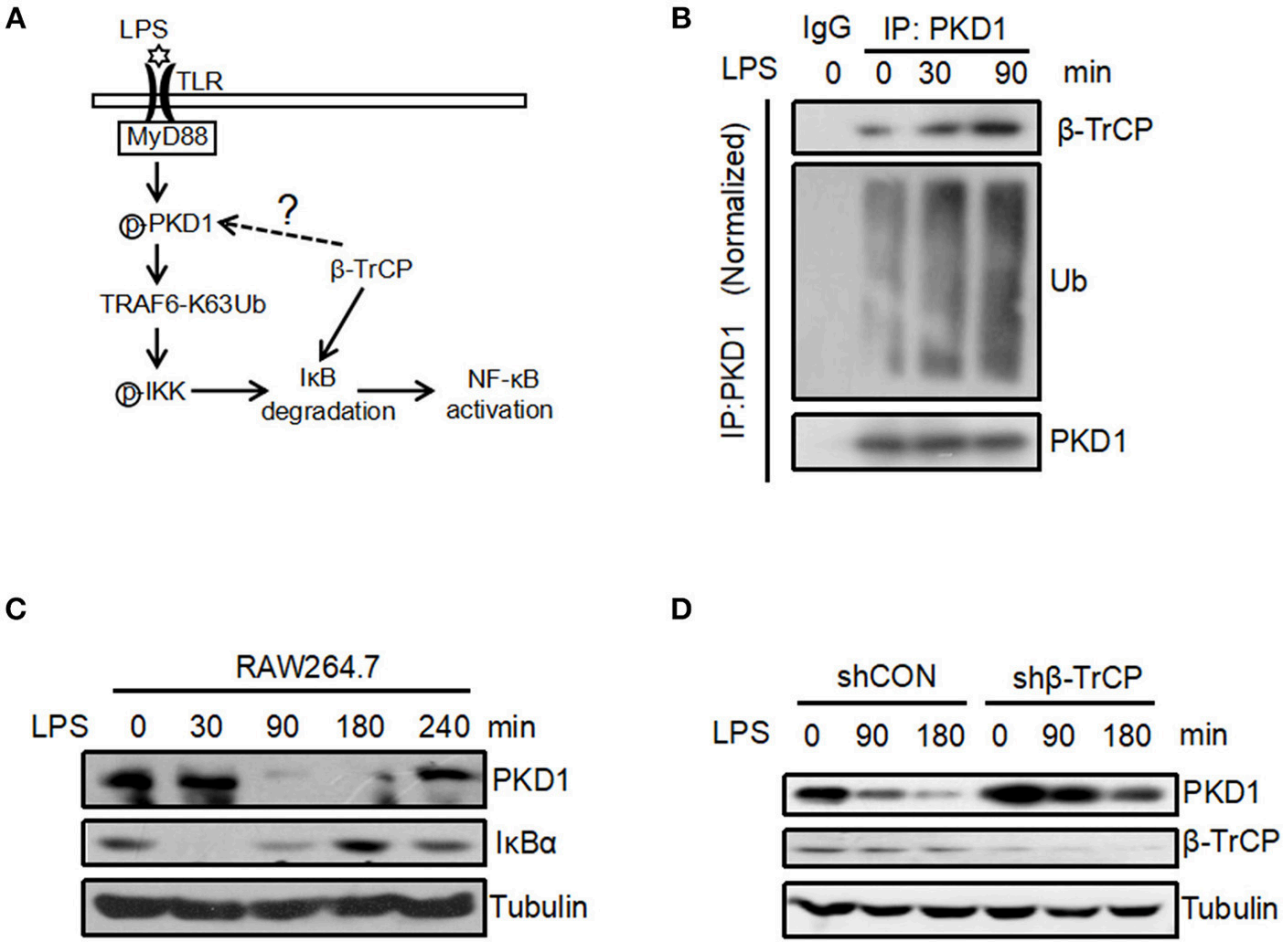
LPS promotes binding of β-TrCP to PKD1 and downregulates PKD1 protein levels. **(A)** The model for LPS-induced inflammatory signaling. **(B)** RAW264.7 cells were treated with LPS (2.5 μg/mL) for 0, 30, 90 min. PKD1 were immunoprecipitated and then immunoblotting was performed using indicated antibodies. **(C)** RAW264.7 cells were stimulated with LPS (2.5 μg/mL) for 0, 30, 90, 180, 240 min. The protein levels of PKD1, IκBα and Tubulin were analyzed by immunoblotting as indicated. **(D)** RAW264.7 cells were transfected with either shCON or shβ-TrCP. Seventy two hours after transfection, cells were stimulated with LPS (2.5 μg/mL) for 0, 90, 180 min. The protein levels of PKD1, β-TrCP and Tubulin were analyzed by immunoblotting as indicated.

Our above findings reveal that β-TrCP interacts with PKD1, and promotes PKD1 ubiquitination and degradation. Interestingly, we noticed that both β-TrCP and PKD1 are involved in LPS induced inflammatory signaling pathway (Figure 4A). Thus, we sought to determine whether the regulation of β-TrCP on PKD1 occurs in LPS inflammatory signaling. To this end, the interaction between β-TrCP and PKD1 in LPS signaling was analyzed. We found that LPS significantly promoted binding of β-TrCP to PKD1 (Figure 4B), and simultaneously upregulated PKD1 ubiquitination (Figure 4B). These data suggest that β-TrCP could interact with and regulate PKD1 during LPS stimulation.

To further study the possible regulation of LPS stimulation on PKD1, we analyzed protein levels of PKD1 in RAW264.7 cells under the conditions of LPS treatment for various times. Using a specific antibody against PKD1, we found that PKD1 protein levels were significantly reduced at 90 min and 180 min stimulation by LPS, whereas IκBα levels gradually increased simultaneously (Figure 4C). Importantly, when β-TrCP was knocked down, LPS-induced downregulation of PKD1 was markedly inhibited (Figure 4D), suggesting that β-TrCP is responsible for PKD1 downregulation in LPS signaling. Collectively, we demonstrate that LPS stimulation can promote binding of β-TrCP to PKD1, and results in PKD1 downregulation.

### Supplement of PKD1 in Cells Inhibits Recovery of IκBα Protein Levels in LPS Signaling

**Figure 5:**
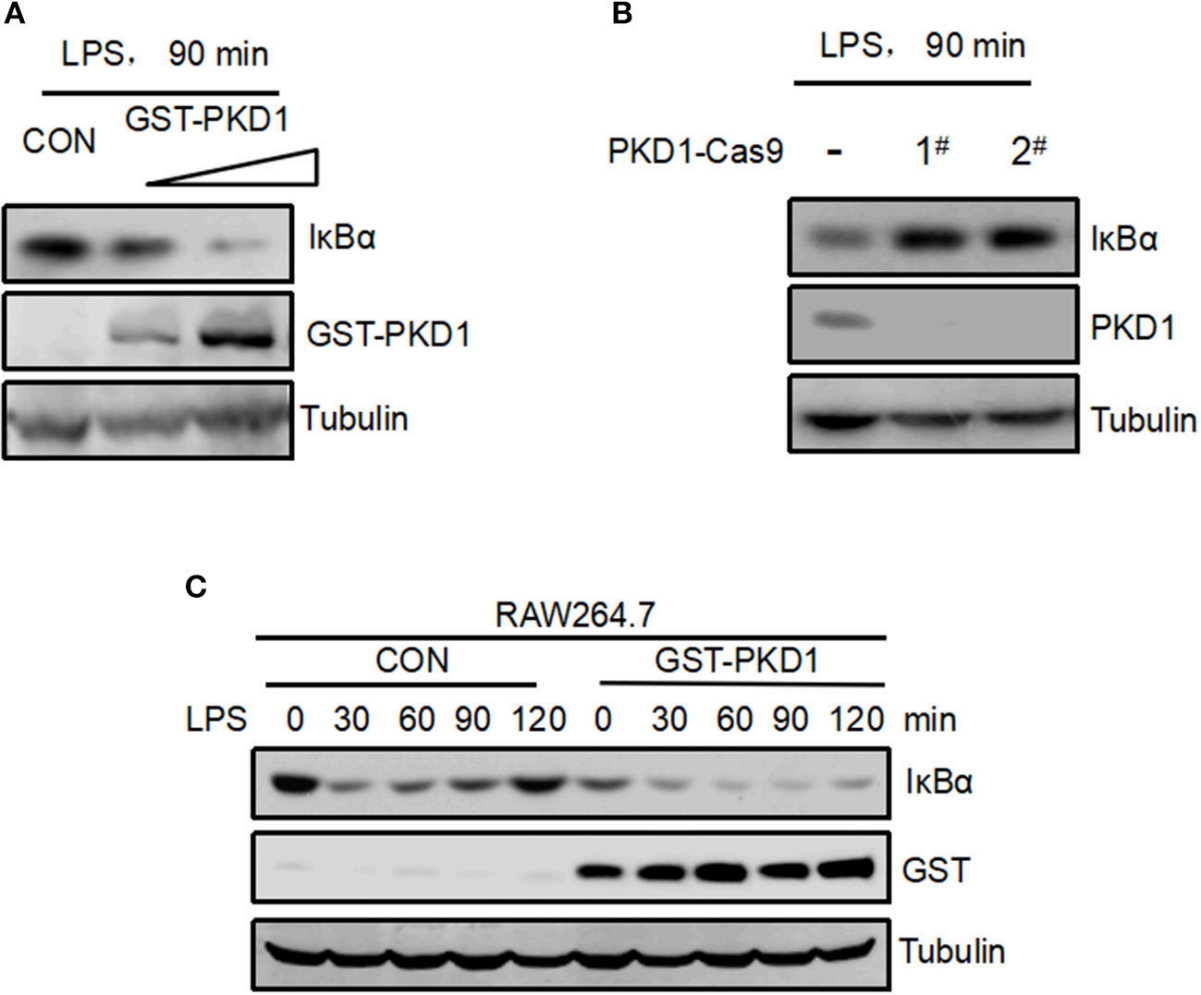
Supplement of PKD1 in cells inhibits recovery of IκBα protein levels in LPS signaling. **(A)** RAW264.7 cells were transfected with increasing does of GST-PKD1. 48 h after transfection, cells were stimulated with LPS (2.5 μg/mL) for 90 min. Protein levels of GST-PKD1, IκBα, and Tubulin were analyzed by immunoblotting as indicated. **(B)** PKD1 in RAW264.7 cells was knocked out by CRISPR–Cas9 genome editing. Two guide RNAs were designed to target PKD1 (1# and 2#). RAW264.7PKD1-Cas9 cells were stimulated with LPS (2.5 μg/mL) for 90 min. Protein levels of PKD1, IκBα, and Tubulin were analyzed by immunoblotting as indicated. **(C)** RAW264.7 cells were transfected with either empty vector or GST-PKD1. Forty eight hours after transfection, cells were stimulated with LPS (2.5 μg/mL) for 0, 30, 60, 90, 120 min. Protein levels of GST-PKD1, IκBα, and Tubulin were analyzed by immunoblotting as indicated.

Interestingly, from the above results we observed a negative correlation between PKD1 and IκBα protein levels in LPS signaling (Figure 4C). It is actually not difficult to understand. It has been clearly demonstrated that LPS stimulation can activate TLR4 signaling and lead to IκBα protein degradation very rapidly. In conjunction with our findings here showing that the upstream signaling molecule PKD1 can be downregulated after LPS stimulation, we speculate that the LPS-TLR4 signaling could be inhibited during the PKD1 downregulation stage, which could result in decrease of IκBα phosphorylation and recovery of IκBα protein levels. That is, PKD1 downregulation during LPS stimulation could contribute to recovery of IκBα protein levels. Next, we try to provide more evidence for this speculation. Given that manipulation of β-TrCP could directly affect IκBα, which impedes the observation of the dynamic changes of IκBα protein levels during LPS stimulation, we took advantage of PKD1 overexpression to supplement PKD1 levels in cells. Firstly, RAW264.7 cells were transfected with increasing dose of PKD1. Cells were then treated with LPS for 90 min. Our data showed that PKD1 overexpression continuously lowered IκBα protein levels in a dose-dependent manner (Figure 5A). Conversely, knockout of PKD1 in RAW264.7 cells by CRISPR–Cas9 genome editing substantially upregulated IκBα protein levels in LPS signaling (Figure 5B). Furthermore, we analyzed the dynamic changes of IκBα protein levels in LPS signaling. The result showed that during LPS stimulation, IκBα protein levels significantly decreased at 30 min time point. After that, IκBα protein levels were gradually recovered (Figure 5C, left panel). When cells were supplemented with PKD1, the recovery of IκBα protein levels was dramatically delayed (Figure 5C, right panel). Collectively, we believe that PKD1 downregulation could contribute to recovery of IκBα protein levels in LPS signaling.

### β-TrCP Negatively Regulates LPS-Induced TRAF6 Ubiquitination and IKK Activation

**Figure 6:**
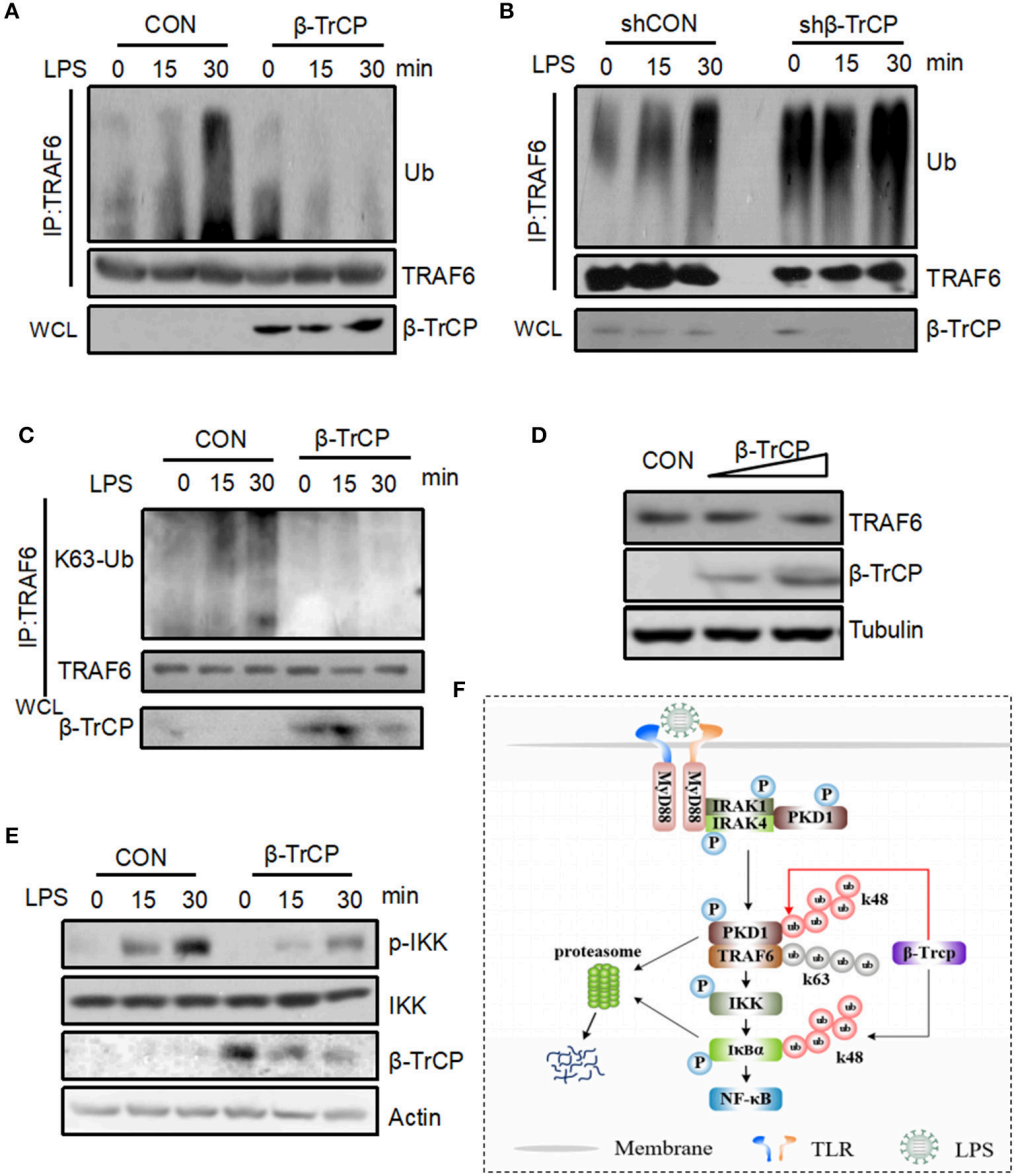
β-TrCP negatively regulates LPS-induced TRAF6 ubiquitination and IKK activation. **(A)** RAW264.7 cells were transfected with β-TrCP. Forty eight hours after transfection, cells were stimulated with LPS (2.5 μg/mL) for 0, 15, 30 min. Endogenous TRAF6 were pulled down, and ubiquitination and protein levels of TRAF6 were analyzed using indicated antibody. **(B)** RAW264.7 cells were transfected with either shCON or shβ-TrCP. Seventy two hours after transfection, cells were stimulated with LPS (2.5 μg/mL) for 0, 15, 30 min. ubiquitination and protein levels of TRAF6 were analyzed as **(A)**. **(C)** RAW264.7 cells were transfected with β-TrCP. Forty eight hours after transfection, cells were stimulated with LPS (2.5 μg/mL) for 0, 15, 30 min. Endogenous TRAF6 were immunoprecipitated, and K63-linked polyubiquitination and protein levels of TRAF6 were analyzed using indicated antibody. **(D)** Total protein levels of TRAF6 were analyzed using indicated antibodies. **(E)** RAW264.7 cells were transfected with or without β-TrCP. Forty eight hours after transfection, cells were stimulated with LPS (2.5 μg/mL) for 0, 15, 30 min. The levels of p-IKK, IKK, β-TrCP, and Actin were analyzed by immunoblotting as indicated. **(F)** Hypothetical model for β-TrCP-mediated regulation of LPS inflammatory signaling pathway. LPS stimulation activates TLR4-PKD1-TRAF6-IKK signaling, which rapidly induces IκBα phosphorylation and subsequent IκBα ubiquitination and degradation mediated by β-TrCP. Finally, NF-κB inflammatory signaling was activated. During LPS stimulation, β-TrCP also interacts with PKD1, which results in PKD1 ubiquitination and degradation, and leads to inhibition of LPS-TLR4-PKD1-TRAF6-IKK signaling. Together, β-TrCP plays both promotion and brake roles in LPS-induced inflammatory signaling.

Given that we have demonstrated that β-TrCP promotes PKD1 downregulation in LPS signaling, we further study whether β-TrCP can regulate the signaling pathway downstream of PKD1 and upstream of IκBα. In LPS signaling, PKD1 activation leads to TRAF6 polyubiquitination and subsequent IKK phosphorylation and activation. Therefore, we analyzed the roles of β-TrCP in regulating TRAF6 ubiquitination and IKK phosphorylation in LPS signaling. Our data showed that β-TrCP overexpression significantly inhibited LPS-stimulated ubiquitination of TRAF6 (Figure 6A). Conversely, knockdown of β-TrCP remarkably promoted TRAF6 ubiquitination induced by LPS (Figure 6B). Furthermore, we found that overexpression of β-TrCP reduced K63-linked polyubiquitination of TRAF6 (Figure 6C), but had no obvious effect on TRAF6 protein levels (Figure 6D), suggesting that β-TrCP inhibits TRAF6 signaling activation. Next, we studied whether β-TrCP could regulate IKK phosphorylation. Using a specific antibody against activated IKK at Ser176/180 site, we found that β-TrCP obviously inhibited LPS-stimulated IKK phosphorylation (Figure 6E). Taken together, our findings demonstrate that β-TrCP negatively regulates LPS-induced TRAF6 K63-linked ubiquitination and IKK activation.

## Discussion

PKD1, as a ubiquitous serine-threonine protein kinase, plays crucial roles in multiple biological processes, including cell growth, adhesion, motility, and angiogenesis (2–5). Stable PKD1 levels in cells are extremely important for normal physiology functions of cells. Dysregulated PKD1 expression could result in the pathogenesis of some cancers. PKD1 could be activated by various stimuli including growth factor (23), oxidative stress (24), and apoptotic signaling (25). Thus far, the regulation of PKD1 expression remains largely unexplored. It has been reported that PKD1 expression is gradually reduced during breast cancer progression, which could be directly associated with hypermethylation of PKD1 promoter (26). Recent studies demonstrated that there is a correlation between the expression of PKD1 and ERα in breast cancer cells (27). However, ubiquitination-mediated regulation of PKD1 remains unknown so far. In this study, we for the first time reveal that ubiquitin E3 ligase β-TrCP regulates PKD1 protein levels in cells. We clearly demonstrated that in LPS signaling β-TrCP can interact with PKD1, which leads to PKD1 downregulation. Furthermore, β-TrCP-mediated downregulation of PKD1 restricts LPS-induced TRAF6 K63-linked polyubiquitination and IKK phosphorylation. Thus, our findings uncover the novel regulation mediated by the β-TrCP-PKD1 axis in LPS inflammatory signaling. In addition, it has been reported that PKD1 can be activated by TLR-mediated MyD88 signaling pathway. Thus, it could be interesting to observe the effects of β-TrCP on PKD1 levels in different TLR ligands-activated signaling pathways in the future.

It has been well-characterized that β-TrCP's substrates contain a consensus DSG(X)_2+n_S degron. The serine residues in this degron could be phosphorylated by certain protein kinases, which results in β-TrCP recognition and subsequent ubiquitination of the substrates (28–30). In this study, we found that PKD1 does possess a potential DSG(X)_2+n_S motif. However, this DSG(X)_2+n_S motif of PKD1 is not important for β-TrCP binding and PKD1 degradation. Through analyzing all conserved XSG(X)_2+n_S motif in various species of PKD1, we finally found the NSG(X)_2+n_S motif of PKD1 is essential for β-TrCP binding and subsequent degradation of PKD1. As a matter of fact, some studies have reported several “atypical” substrates of β-TrCP, including STAT1 (19), Weel (20), and GHR (21), which do not have the classic DSG(X)_2+n_S motif. Here, our study uncovers a new “atypical” degron in PKD1 for β-TrCP-mediated degradation.

Prevailing thought holds that β-TrCP is a positive regulator for TLRs-NF-κB inflammatory signaling, because β-TrCP induces IκBα degradation and NF-κB release. In this study, we demonstrate that β-TrCP could be a negative regulator for upstream signaling of TLRs-NF-κB pathway. LPS stimulation activates TLR4-PKD1-TRAF6-IKK-IκBα signaling, which leads to β-TrCP-mediated ubiquitination and degradation of IκBα rapidly. Unexpectedly, we find that during LPS stimulation β-TrCP also binds to PKD1, which results in PKD1 ubiquitination and degradation. PKD1 downregulation inhibits further activation of LPS-PKD1-TRAF6-IKK signaling (Figure 6F). As observed in this study, β-TrCP-mediated PKD1 downregulation inhibits TRAF6 K63-linked polyubiquitination and subsequent IKK phosphorylation. Also, we observed that PKD1 protein levels are negatively correlated with IκBα levels in LPS signaling, and supplement of PKD1 in cells significantly delays recovery of IκBα protein levels. Taken together, we think that β-TrCP plays both promotion and brake roles in LPS-induced inflammatory signaling. The dual effects of β-TrCP may provide the balance for LPS inflammatory signaling. Based on the dual effects of β-TrCP, we focus on studying LPS-IKK upstream signaling, rather than analyzing cytokines production downstream of IκBα signaling.

In summary, we demonstrate that β-TrCP specifically targets PKD1 for K48 ubiquitination and degradation in an “atypical” binding manner. Furthermore, we uncover that during LPS stimulation β-TrCP can interact with PKD1 and promotes PKD1 downregulation, which in turn restricts TRAF6-IKK signaling upstream of IκBα in LPS inflammatory pathway. Our findings reveal that β-TrCP is an important negative regulator for upstream signaling of IκBα in LPS signaling, and therefore renew our understanding of the roles of β-TrCP in TLRs inflammatory signaling.

## Author Contributions

HZ, JW, and JL designed the research. JL, YY, JX, KX, and YX performed the experiments. HZ and JL wrote the manuscript. YX, LZ, and TG provided expertise and advice. HZ supervised the project.

### Conflict of Interest Statement

The authors declare that the research was conducted in the absence of any commercial or financial relationships that could be construed as a potential conflict of interest.
